# Exploring the role and mechanism of Fuzi decoction in the treatment of osteoporosis by integrating network pharmacology and experimental verification

**DOI:** 10.1186/s13018-023-03842-1

**Published:** 2023-07-18

**Authors:** Fudong Li, Chuan Guo, Shikai Zhang, Bing Zheng, Kaiqiang Sun, Jiangang Shi

**Affiliations:** 1 Department of Orthopaedic Surgery, Spine Center, Shanghai Changzheng Hospital, Naval Medical University, Shanghai, 200003 China; 2 Orthopedic Research Institute, Department of Orthopedics, West China Hospital, Sichuan University, Chengdu, 610065 China; 3 Department of Orthopaedic Surgery, Shanghai Kaiyuan Orthopaedic Hospital, Shanghai, 200129 China

**Keywords:** Fuzi decoction, Osteoporosis, Network pharmacology, Osteoclast, NF-κB pathway

## Abstract

**Background:**

Fuzi decoction (FZD), a traditional Chinese medicine formula, was used to treat musculoskeletal diseases by warming channels, strengthening yang and dispelling pathogenic cold and dampness. In clinical practice, FZD has been used to treat rheumatoid arthritis and osteoarthritis. It alleviated osteoarticular disorders through ameliorating the degradation of cartilage and improving meniscal damage in osteoarthritis, while its roles and mechanisms in the treatment of bone loss diseases remain unclear. This study aims to investigate the underlying mechanisms of FZD in treating osteoporosis using an integrative method of network pharmacology and experimental study.

**Methods:**

In this study, network pharmacology was used to predict the core targets and potential pathways of the bioactive ingredients of FZD to attenuate osteoporosis. Molecular docking was performed to evaluate the interactions between core compounds and key targets. In addition, both cell and animal experiments were carried out to validate the role and potential mechanism in treating osteoporosis.

**Results:**

In the present study, data revealed that kaempferol, beta-sitosterol, stigmasterol, fumarine, and (+)-catechin may be the primary bioactive ingredients of FZD in the treatment of osteoporosis, which were closely associated with the osteoporosis-related targets. And the KEGG results indicated that the NF-κB pathway was closely associated with the function of FZD in treating osteoporosis. In addition, in vivo demonstrated that FZD ameliorated osteoporosis. In vitro experiments showed that the pro-apoptotic factors indicators including CASP3 and BAX were decreased by FZD and the anti-apoptotic factor BCL2 was increased by FZD. In addition, FZD significantly suppressed the osteoclast differentiation in culture and the expression levels of osteoclast-related genes including TRAF6, CTSK, and MMP9. And the NF-κB pathway was confirmed, via in vitro experiment, to be involved in osteoclast differentiation.

**Conclusions:**

This study demonstrated that FZD played a pivotal role in suppressing the osteoclast differentiation via regulating the NF-κB pathway, indicating that FZD could be a promising antiosteoporosis drug and deserve further investigation.

**Supplementary Information:**

The online version contains supplementary material available at 10.1186/s13018-023-03842-1.

## Background

Osteoporosis, a major health problem worldwide, is characterized by the loss of bone mass and the destruction of bone microstructure that can increase bone fragility [1]. It was reported that an estimated $13.8 billion was spent on osteoporotic fractures, one of the most common complications of osteoporosis, in the USA in 1995 [2]. A study revealed that 43,000 deaths were caused by osteoporosis-related complications in the European Union in 2010 [3]. Bone homeostasis is regulated by osteoblasts (OBs) that are responsible for bone formation and osteoclasts (OCs) that are responsible for bone resorption. The excessive activation of OCs can lead to the imbalance between OCs and OBs, causing osteolytic bone diseases such as osteoporosis [4]. Therefore, inhibiting the excessive activation of OCs is essential in preventing and treating osteoporosis. Prophylaxis and early diagnosis are essential in the prevention and treatment of osteoporosis and osteoporotic fractures. Anti-osteoporotic medications are desirable for treating and preventing osteoporosis. Biphosphonates were commonly used to increase bone density and reduce fracture risk [5]. It was reported that Alendronate reduced the risk of osteoporotic fractures in a randomized controlled trial [6]. However, various adverse events in patients who were treated with the above medications have been reported, which included hypocalcaemia, secondary hyperparathyroidism, musculoskeletal pain, osteonecrosis of the jaw and ocular events [7]. Therefore, more effective and safer treatment is urgently needed.

Traditional Chinese medicine (TCM) has gained popularity in recent years as a treatment for musculoskeletal disorders due to its mild side effects and obvious benefits. Several researchers have recently reported the application of TCM in treating osteoporosis [8, 9]. Previous studies have reported that some TCM components, such as Danggui, Chuanxiong, Xuduan, and Duzhong, were associated with the treatment of bone diseases, such as osteopenia and osteoporosis [10–13]. It was recorded, approximately 1800 years ago, in the famous medical book “Treatise on Febrile and Miscellaneous Diseases” for the first time that Fuzi decoction (FZD) could be used to treat osteoarticular pain. FZD is composed of monarch drug Fuzi (the rhizome of Aconitum carmichaeli Debeaux) and minister drugs that function as the assistants of Fuzi, which includes Fuling (the dried sclerotum of the fungus), Renshen (the root and rhizome of Panax ginseng C.A.Mey), Baizhu (the dried rhizome of Atractylodes macrocephala Koidz) and Shaoyao (the dried rhizome of Paeonia lactiflora Pall). The plant names have been checked with http://www.theplantlist.org. According to the records of Treatise on Febrile and Miscellaneous Diseases, the principal role of FZD is ameliorating the osteoarticular pain through warming channels, strengthening yang and dispelling pathogenic cold and dampness. In addition, it was reported that the monarch drug Fuzi could exert bone-protective effects because of its anti-inflammatory, analgesic, and chondroprotective roles [14, 15]. The study conducted by Tong and colleagues demonstrated that Fuzi could alleviate mono-iodoacetate-induced osteoarthritis through ameliorating cartilage degeneration and improving the bone density in rats [15]. A recent study also discovered that FZD ameliorated the degradation of cartilage in osteoarthritis [16]. In their study, the anabolism was promoted and the catabolism was suppressed in osteoarthritis rats by FZD; and the decreased expression of aggrecan and the increased levels of MMP13 and ADAMTS5 in IL-1β-treated chondrocytes were significantly restored after the treatment of FZD [16]. The data in their study also revealed that FZD could alleviate the joint pain and improve meniscal damage in osteoarthritis patients [16]. The above evidence demonstrated that FZD could be a potential treatment strategy for osteoarticular diseases. It is worth noting that osteoporosis has also been proved to be closely associated with the degradation of extracellular matrix caused by excessive osteoclast activation [17]. Theoretically, according to the roles of FZD or Fuzi in treating osteoarthritis, FZD might have a therapeutic effect on osteoporosis. However, there are few literatures about the role and molecular mechanism of FZD in treating osteoporosis.

Network pharmacology, a method of drug research, has been used to analyze the relationship among biological systems, drugs and diseases [18]. The concept of network pharmacology was initially proposed by Andrew L. Hopkins in 2007 [19]. The novelty of this approach is that it employs systems biology, network analysis, connectivity, and redundancy [19]. Network pharmacology can be used as a potent research approach to transform TCM from traditional experience-based medicines to modern medicines based on objective evidence. Based on the concept of “network target, multi-components”, network pharmacology can discover the therapeutic effects of TCM on various diseases and elucidate the molecular mechanism of TCM formulae in systematic view. Until now, many studies based on the network pharmacology have been reported for investigating the effects of TCM on osteoporosis. In a study of network pharmacology, Qin et al. reported that Erzhi formulacouldrelieve the bone loss induced by ovariectomy (OVX) in mice [3]. And the study revealed that Erzhi formula inhibited bone resorption through decreasing the osteoclast maturation [3]. In addition, Han et al. revealed that Curculigoside A could suppress the expression level of genes related to matrix metalloproteinase (MMP) in rheumatoid arthritis and osteoporosis through methods of network pharmacology and experimental verification [20].

In this present study, network pharmacology and experimental validation were applied to explore the role and potential mechanism of FZD in the treatment of osteoporosis. This study provided theoretical basis and experimental evidence for this strategy.

## Methods

### Chemical components database

FZD consists of five traditional Chinese herb medicines including Fuzi, Fuling, Renshen, Baizhu, and Shaoyao. The bioactive compounds of each herb in FZD were obtained from the Traditional Chinese Medicine Systems Pharmacology Database and Analysis Platform (TCMSP, https://old.tcmsp-e.com/tcmsp.php). Substances with oral bioavailability (OB) ≥ 30% and drug-likeness (DL) ≥ 0.18 were identified as pharmacological compounds [21, 22].

### Prediction of potential targets of FZD during the treatment of osteoporosis

Through the keyword “osteoporosis”, osteoporosis-related targets were collected from five databases: the GeneCards database (https://www.genecards.org/), the Online Mendelian Inheritance in Man (OMIM) database (https://www.omim.org/), the Therapeutic Target Database (TTD) (http://db.idrblab.net/ttd), the PharmGKB database (https://www.pharmgkb.org/), and the DrugBank database (https://go.drugbank.com/). The databases were merged and the duplicate targets were removed. Then common targets were selected through intersection of the active ingredient targets of FZD and osteoporosis-related targets. The Venn diagram was plotted using the R software (R version R-4.2.0). It was considered that these common targets were the potential targets of FZD for treatment of osteoporosis.

### Protein–protein interaction (PPI) analysis

To reveal the relationships among the common targets, the STRING database was applied to obtain protein–protein interaction data. Then the PPI network of the common target proteins were constructed by Cytoscape software (version 3.9.1). A network topology analysis was performed to calculate the degree of each node through Network Analyzer (a Cytoscape plugin). And core targets were screened using Cytoscape. In the PPI network, the minimum required interaction score was medium confidence (0.4) and the disconnected nodes were hided in the network.

### Gene ontology (GO) and Kyoto encyclopedia of genes and genomes (KEGG) enrichment analysis

To further understand the possible biological functions and metabolic pathways involved in these genes, the Gene Ontology (GO) enrichment and the Kyoto encyclopedia of genes and genomes (KEGG) pathway enrichment analysis were performed with R software (R version R-4.2.0). The top 30 relevant KEGG pathways and the top 20 relevant GO enrichment results (*p* < 0.05) were shown in the plots.

### Molecular docking

To clarify the molecular mechanism, we analyzed the binding affinity, binding sites, and interactions between the core active compounds ((+)-catechin, beta-Sitosterol, Frutinone A, Fumarine, Kaempferol, and Stigmasterol) and the predicted core target (DPP4, PGR, PTGS1, PTGS2, and RXRA). Ligand structures were obtained from PubChem database (https://pubchem.ncbi.nlm.nih.gov/), and Chem3D was used to minimize the energy of ligands. The PDB files of the proteins were retrieved from the PDB database (http://www.rcsb.org/pdb). Then water and ions of the structures were deleted by PyMOL software (version 1.7.1.0). The PDB files were transformed into the PDBQT format by the AutodockTools software (version 1.5.6) that can find the active pockets of the core proteins. The Autodock Vina-1.1.2 was applied to perform the molecular docking and to determine the binding energies. The molecules and proteins can form a stable complex if the binding energy ≤  − 5.0 kcal mol^−1^ and the RMSD value < 2.00. A binding value ≤  − 7.0 kcal mol^−1^ reveals a strong binding of the molecules to the receptor. The PyMOL software was used to visualize the results of molecular docking.

### Preparation of FZD

Based on standard protocols, herbs in FZD were purchased from Shanghai Changzheng Hospital, which included Fuzi (15 g), Fuling (9 g), Renshen (6 g), Baizhu (12 g), Shaoyao (9 g). The drugs were soaked in 10 volumes of water for 30 min and boiled for 60 min. The decoction was filtered by medical gauze. Then the remaining herbs were boiled in 8 volumes of water and the process was repeated. The filtrates were pooled and concentrated to 1 g/ml and stored at 4 °C.

### Animals model

The animal experiment was approved by the Ethical Committee of Shanghai Changzheng Hospital. Female Sprague–Dawley rats (6–8 weeks) were purchased from the Experimental Animal Center of the Zhejiang Academy of Medicinal Sciences (Hangzhou Medical College, Hangzhou, China) and randomly divided into sham, sham + FZD, OVX, and OVX + FZD groups. In rats anesthetized with isoflurane, bilateral ovariectomy was performed to induce osteoporosis in OVX and OVX + FZD groups. In the sham and sham + FZD group, the ovaries of rats were exposed but not resected. The rats in sham + FZD group and OVX + FZD group were given FZD (5.4 g/kg/day) by gavage twice daily (morning and evening) for 35 days as previous report [16], and the rats in the sham group and the OVX group were given the same volume of saline.

### Obtaining osteoclasts from bone marrow-derived macrophages (BMMs)

Rats (6–8 weeks) were sacrificed through cervical dislocation after isofluorane-induced anesthesia. Then rats were sterilized with 75% alcohol immersion for five minutes. Bone marrow cells were harvested from the femur through flushing with phosphate buffer solution (PBS). The BMMs were cultured with alpha-minimum essential medium (α-MEM) containing 50 ng/ml M-CSF. Then 50 ng/ml receptor activator of NF-kB ligand (RANKL) was used to treat BMMs to induce osteoclast differentiation.

### Preparation of FZD-containing serum (FCS)

For in vitro experimental verification, the FZD-containing serum (FCS) and blank serum (BS) were prepared using TCM serum pharmacology methods. Rats in FCS group or in BS group received FZD (5.4 g/kg/day) or the same volume of sterile distilled water for seven consecutive days, respectively. Blood was collected via cardiac puncture. After centrifugation (1000×*g* for 10 min, at 4 °C), the serum was filtered through a 0.22 μm membrane, which were then incubated at 56 °C for half an hour. Both the FCS and the BS were stored at − 80 °C.

### Cell counting kit-8 (CCK-8)

To evaluate the potential cytotoxicity of, the Cell counting kit-8 (CCK-8) analysis was carried out. The CCK-8 analysis was carried out according to the manufacturer instruction (Dojindo, Japan). Primary BMMs from bone marrow were directly seeded into 96-well cell plates. FZD was added to each well at concentration gradient after replacing the complete medium with serum-free medium. After 24 h, cells were rinsed and the CCK-8 solution was added into the plates. Absorbance density (OD) values at 480 nm measured through a microplate reader (Bio-Tek, USA) was used to evaluate the cell viability. Cell viability (%) = (OD of test group/OD of control group) × 100%.

### Micro-computed tomography (micro-CT) analysis

The femurs were carefully collected after perfusion with 4% paraformaldehyde. A micro-computed tomography (micro-CT) system (Skyscan1176; Bruker, USA) was used to image the femora with the following parameters: voltage of 50 kV, current of 450–500 μA, and isotropic resolution of 9 μm. The images were built with NRecon software (Bruker microCT, Kontich, Belgium). The bone mineral density (BMD), the relative bone volume (bone volume/tissue volume, BV/TV), bone surface density (bone surface/tissue volume, BS/TV), connectivity density, total porosity, trabecular number, trabecular separation, and trabecular thickness of the micro-CT results were analyzed.

### Cytoskeletal F-Actin podosome belt staining

MCSF and RANKL were used to induce osteoclast differentiation. BMMs were treated with FCS or PBS until the formation of multinucleated osteoclasts. Then cells were fixed with 4% paraformaldehyde and treated with 0.5% Triton X-100 for 5 min. Cells were then incubated with Phalloidin-iFluor 488 Reagent (CA1610, Solarbio, Shanghai, China) in 1% BSA-PBS for 30 min at room temperature. Nuclear counterstaining was carried out using DAPI. The images of F-actin belt was captured with fluorescence microscopy and quantified by Image J software.

### Tartrate-resistant acid phosphatase (TRAP) staining analysis

To identify osteoclasts, TRAP staining was carried out. BMMs were used for TRAP staining when the fused cells could be observed with light microscope (Nikon, Tokyo, Japan). The osteoclasts were fixed in 4.0% paraformaldehyde for 20 min before stained with TRAP working solution for half an hour at 37 °C. TRAP working solution was prepared according to the manufacturer guidelines (Servicebio, G1050-50T). TRAP-positive (TRAP^+^) osteoclasts were imaged with light microscopy and then counted with Image J software. TRAP^+^ cells with more than 3 nuclei were regarded as osteoclasts. In addition, TRAP staining was performed on the femoral sections. Paraffin embedded sections were dewaxed and rehydrated. Then the sections were stained using the TRAP staining kits (Servicebio, G1050-50T). And the slices were imaged with the light microscope (Nikon, Tokyo, Japan).

### Western blot

Total cellular proteins were extracted with RIPA lysis buffer (Beyotime, China), which were then quantified by the bicinchoninic acid (BCA) kit (Beyotime, China). SDS-PAGE was used to separate proteins that were then transferred to polyvinylidene fluoride (PVDF) membranes (Millipore, Billerica, MA, USA). The PVDF membrane was blocked with skimmed milk for 2 h and then incubated with primary antibodies and secondary antibodies. The following antibodies were used: GAPDH (Cell signaling technology, USA, #5174), CASP3 (Abcam, UK, ab184787), BAX (Abcam, UK, ab182733), BCL2 (Abcam, UK, ab182858), MMP9 (Abcam, UK, ab283575), TRAF6 (Abcam, UK, ab33915), Cathepsin K (CTSK) (Abcam, UK, ab187647), p-p65 (Cell signaling technology, USA, #3033), p65 (Cell signaling technology, USA, #8242), NFATC1 (Cell signaling technology, USA, #8032), c-FOS (Abcam, UK, ab222699).

### Real-time quantitative PCR

Total RNA was extracted by the HiPure Total RNA Mini Kit (Magen, China). Real-time PCR was carried out using ChamQ TM Universal SYBR qPCR Master Mix. The levels of mRNA were presented with relative fold. The sequences of primers are provided in Table 1.

**Table 1:** The primer sequences in the present study

Gene	Forward (5′–3′)	Reverse (5′–3′)
CASP3	GGAACGCGAAGAAAAGTG	ATTTTGAATCCACGGAGGT
BAX	GAGGTCTTCTTCCGTGTGG	GATCAGCTCGGGCACTTT
BCL2	AGGAACTCTTCAGGGATGG	GCGATGTTGTCCACCAG
TRAF6	AGCATCTTCCCGGTTTG	GCTGGCGATTTTGTGTTT
CTSK	CGTATGTGGGGCAGGAT	TCTTCAGGGCTTTCTCGTT
MMP9	CGCTGGGCTTAGATCATT	TGCTGGATGCCTTTTATGT
NFATc1	GGCAACACGAGACCCAA	CGTCTTCCACCTCCACATC
c-FOS	CGAGGGGTTCCCGTAGA	ACTCCATGCGGTTGCTTT
GAPDH	CCTTCCGTGTCCCCACT	GCCTGCTTCACCACCTTC

### Statistical analysis

Experiments were performed for three times. All the quantitative data in the present study were expressed as the mean ± SD. Multiple group comparisons were carried out with ANOVA. Graphpad prism (version 8) and SPSS (version 24.0) were used to perform statistical analyses. *p* < 0.05 was statistically significant.

## Results

### Bioactive ingredients of Fuzi decoction and target prediction

The five herbs of FZD, which include Fuzi, Fuling, Dangshen, Baizhu, and Shaoyao, have their bioactive ingredients that were collected if the OB was ≥ 30% and the DL ≥ 0.18. A total of 77 active components of FZD (Baizhu: 7; Fuling: 15; Fuzi: 21; Renshen: 21; Shaoyao: 13) were obtained (Additional file 1). The targets of the herbs were provided in the Additional file 2. A total of 108 targets of FZD were obtained after removing duplications.

### Targets of osteoporosis and the common targets between FZD and osteoporosis

**Fig. 1:**
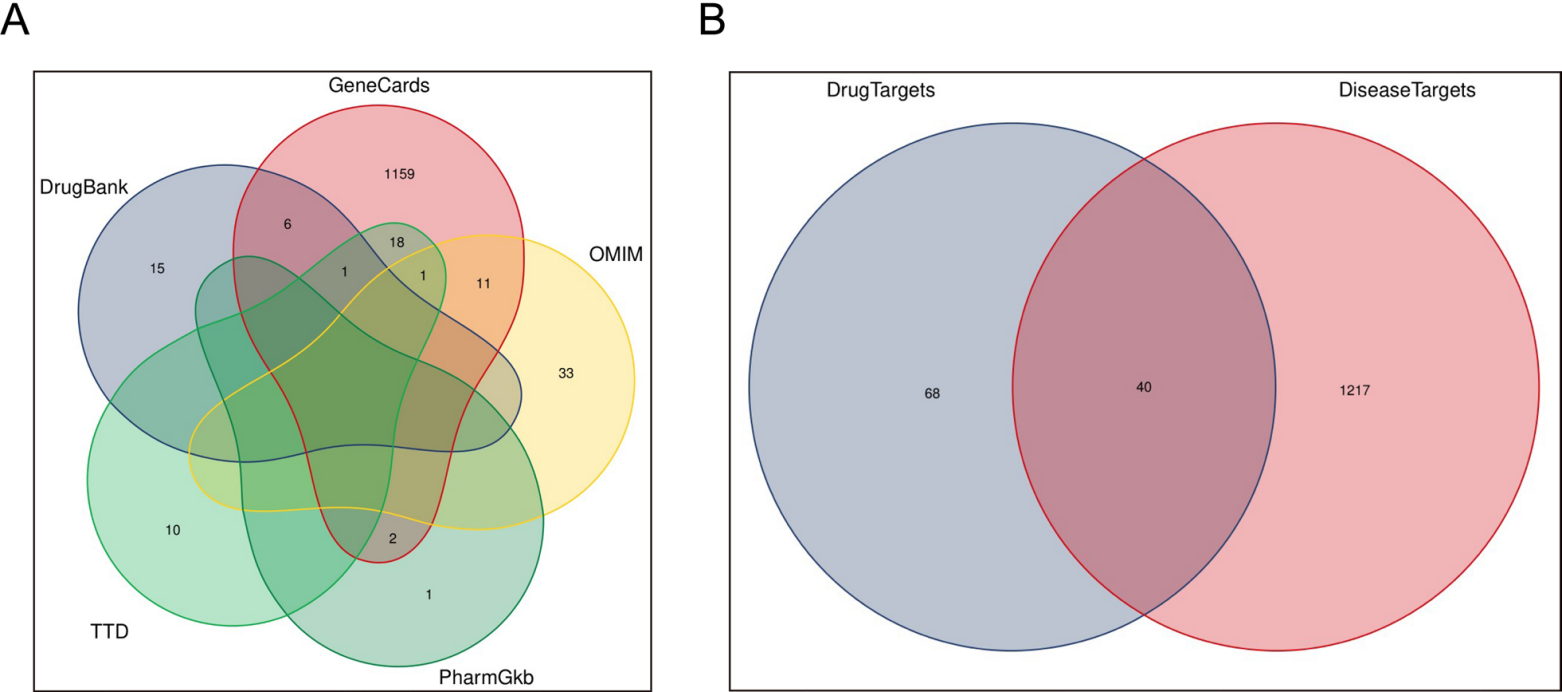
Selection of the targets of osteoporosis and the common targets between FZD and osteoporosis. **A** The number of genes related to osteoporosis were retrieved from five databases. **B** The intersection between targets of FZD and genes of osteoporosis

The gene targets of osteoporosis were retrieved from five databases: GeneCards, OMIM, PharmGkb, DrugBank, and TTD. Initially, 22 targets were obtained from the DrugBank database, 1198 targets from the GeneCards database, 45 targets from the OMIM database, 3 targets from the PharmGkb database, 29 targets from the TTD database (Fig. 1A, Additional file 3). A total of 1257 targets that were closely associated with osteoporosis were obtained after removing duplicates (Fig. 1A). The intersection between targets of FZD and genes of osteoporosis was also displayed as Venn diagram. A total of 40 common targets were identified, which were closely associated with both the FZD ingredients and the osteoporosis (Fig. 1B).

### Identification of the core targets and key ingredients of FZD in treating osteoporosis

**Fig. 2:**
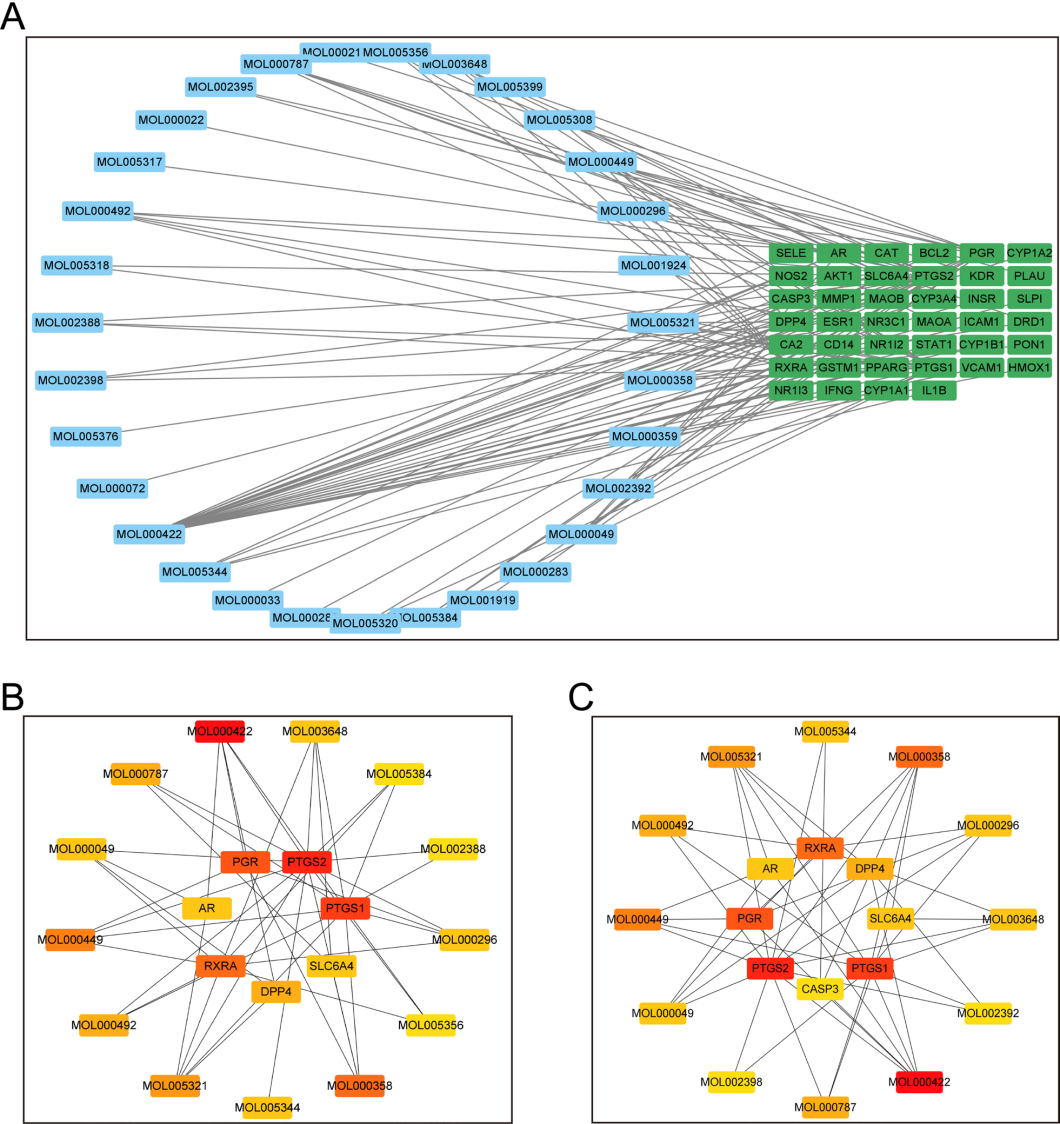
The core targets and key ingredients of FZD in treating osteoporosis. **A** The network between the common targets and the ingredients. **B** The core targets and key ingredients selected by degree. **C** The core targets and key ingredients selected by MCC. The darker the color, the more important the term

Then, to clarify the interaction between the active ingredients of FZD and osteoporosis-related targets, an ingredients-targets network was developed through Cytoscape software, which consists of 71 nodes and 107 edges (Fig. 2A). Core bioactive ingredients and core targets were identified through the degree and the MCC methods using cytoHubba plug-in. Seven core targets and 13 core ingredients were identified. The core targets selected by the degree included PTGS2, PTGS1, PGR, RXRA, DPP4, AR and SLC6A4 (in order of decreasing degree score) (Fig. 2B). The core targets selected by the MCC included PTGS2, PTGS1, PGR, RXRA, DPP4, SLC6A4, AR, and CASP3 (in order of decreasing MCC score) (Fig. 2C). MOL000422 (kaempferol), MOL000358 (beta-sitosterol), MOL000449 (Stigmasterol), MOL005321 (Frutinone A), MOL000787 (Fumarine), MOL000492 ((+)-catechin), MOL005344 (ginsenoside rh2), MOL000049 (3β-acetoxyatractylone), MOL000296 (hederagenin), MOL003648 (Inermin), MOL005356 (Girinimbin), MOL005384 (suchilactone), and MOL002388 (Delphin_qt) were compounds selected by degree and presented in order of decreasing degree score (Fig. 2B). And MOL000422 (kaempferol), MOL000358 (beta-sitosterol), MOL000449 (Stigmasterol), MOL005321 (Frutinone A), MOL000492 ((+)-catechin), MOL000787 (Fumarine), MOL003648 (Inermin), MOL000296 (hederagenin), MOL000049 (3β-acetoxyatractylone), MOL005344 (ginsenoside rh2), MOL002398 (Karanjin), and MOL002392 (Deltoin) were core ingredients selected by MCC method (Fig. 2C). The top 20 entries selected by degree or MCC were presented in Table 2.

**Table 2:** The top 20 entries selected by degree or MCC

Rank	Entries by degree (score)	Type or name	Entries by MCC (score)	Type or name
1	MOL000422 (25)	Kaempferol	MOL000422 (25)	Kaempferol
2	PTGS2 (20)	Gene	PTGS2 (20)	Gene
3	PTGS1 (15)	Gene	PTGS1 (15)	Gene
4	PGR (12)	Gene	PGR (12)	Gene
5	RXRA (8)	Gene	MOL000358 (8)	Beta-sitosterol
6	MOL000358 (8)	Beta-sitosterol	RXRA (8)	Gene
7	MOL000449 (7)	Stigmasterol	MOL000449 (7)	Stigmasterol
8	MOL005321 (6)	Frutinone A	MOL005321 (6)	Frutinone A
9	MOL000787 (5)	Fumarine	DPP4 (5)	Gene
10	MOL000492 (5)	(+)-catechin	MOL000492 (5)	(+)-catechin
11	DPP4 (5)	Gene	MOL000787 (5)	Fumarine
12	MOL005344 (4)	Ginsenoside rh2	MOL003648 (4)	Inermin
13	MOL000049 (4)	3β-acetoxyatractylone	SLC6A4 (4)	Gene
14	AR (4)	Gene	MOL000296 (4)	hederagenin
15	MOL000296 (4)	hederagenin	AR (4)	Gene
16	SLC6A4 (4)	Gene	MOL000049 (4)	3β-acetoxyatractylone
17	MOL003648 (4)	Inermin	MOL005344 (4)	Ginsenoside rh2
18	MOL005356 (3)	Girinimbin	CASP3 (3)	Gene
19	MOL005384 (3)	Suchilactone	MOL002398 (3)	Karanjin
20	MOL002388 (3)	Delphin_qt	MOL002392 (3)	Deltoin

### The establishment of protein–protein interaction (PPI) network and core proteins selected from the PPI network

**Fig. 3:**
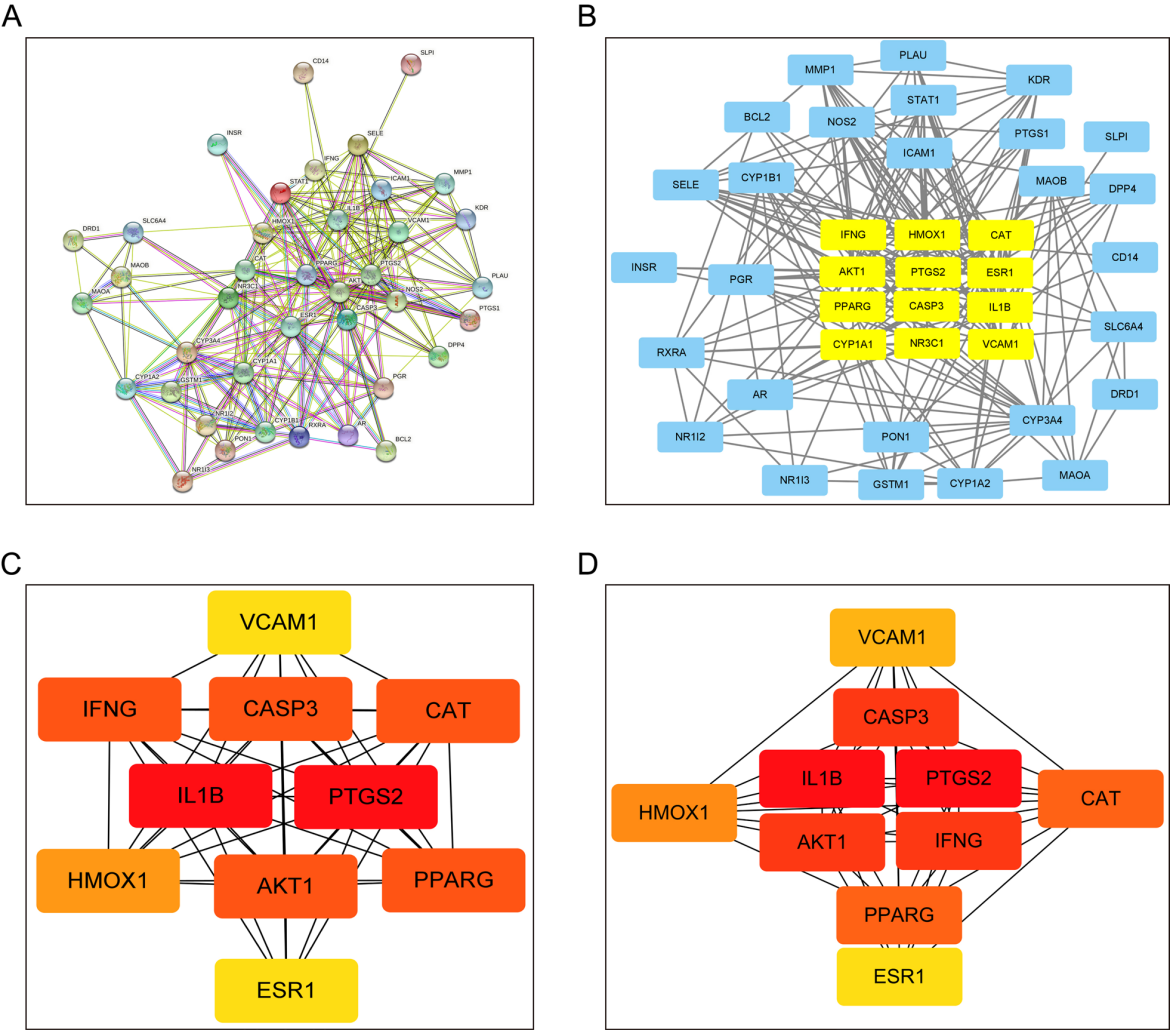
The PPI network and the core proteins selected from the PPI network. **A** The PPI network obtained using the STRING database. **B** The potential core targets of FZD in treating osteoporosis were selected using CytoNCA. **C**, **D** The core targets selected by degree or MCC. The darker the color, the more important the term

To further analyze the targets, a protein–protein interaction (PPI) network was performed through STRING database (http://string-db.org). The targets that were not associated with other targets in the network were hidden. The result of PPI network which include 40 nodes and 235 edges was shown (Fig. 3A). In addition, the potential core targets of FZD in the treatment of osteoporosis were selected using CytoNCA, which included IFNG, HMOX1, CAT, AKT1, PTGS2, ESR1, PPARG, CASP3, IL1B, CYP1A1, NR3C1, and VCAM1 (Fig. 3B). Then the top 10 targets selected through the degree and the MCC methods were presented in Fig. 3C, D, respectively. As can be seen, the key targets that were closely associated with the role of FZD in the intervention of osteoporosis included IL-1B, PTGS2, AKT1, CASP3, PPARG, CAT, IFNG, ESR1, HMOX1, and VCAM1 (Fig. 3C, D). The results showed that the core targets selected by degree or MCC were identical, which were also included in the key targets selected by CytoNCA.

### GO enrichment and KEGG pathway analysis

**Fig. 4:**
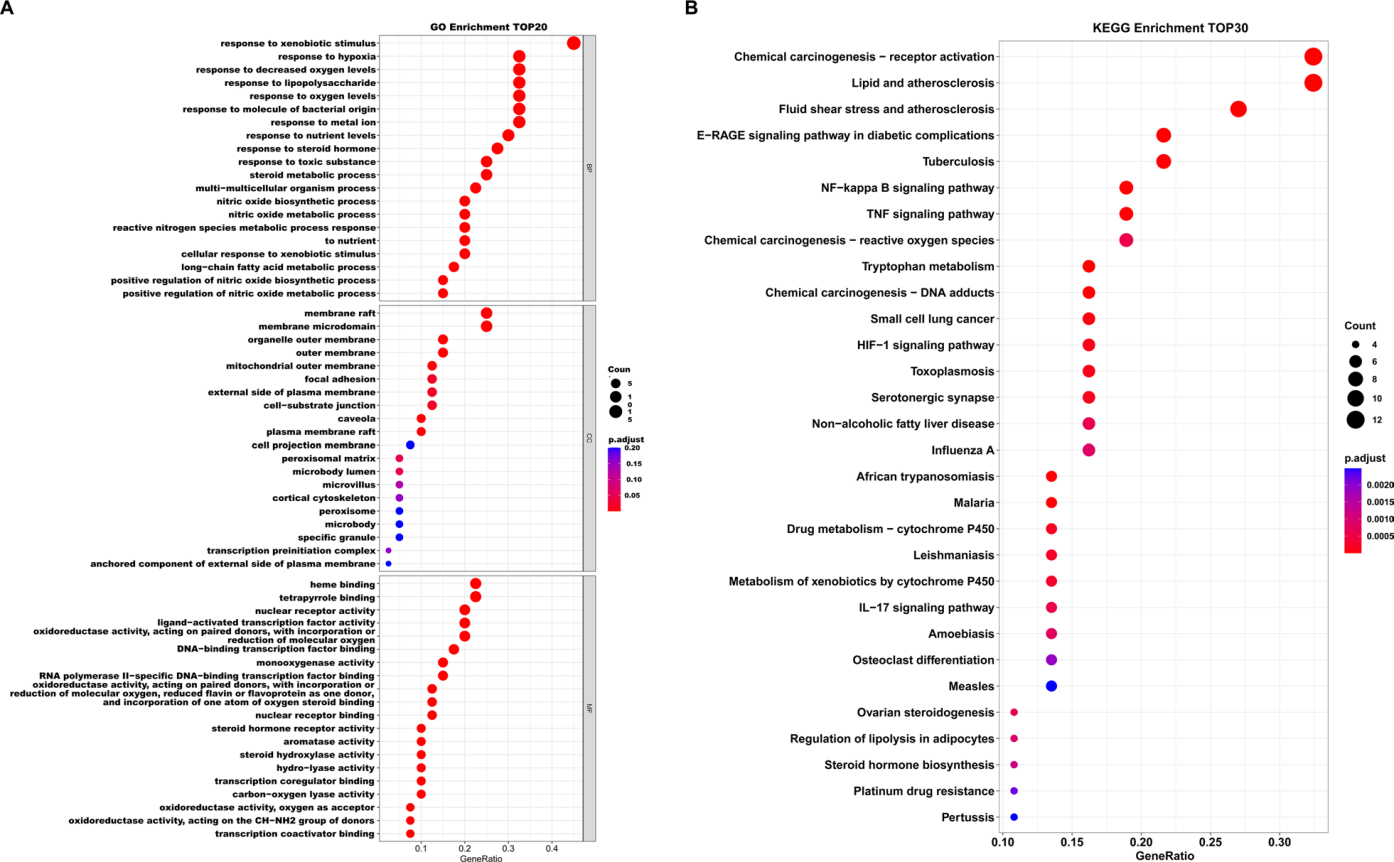
GO enrichment and KEGG pathway analysis. **A** The results of GO enrichment. **B** The results of KEGG analysis

To associate the gene expressions with biological processes, Gene Ontology (GO) enrichment analysis was carried out based on the common targets between FZD and osteoporosis, which included 1275 entries in biological progress (BP), 12 entries in cellular components (CC) and 116 entries in molecular functions (MF) (Additional file 4). In addition, the top 20 GO terms of BP, CC, and MF were shown in the bubble diagram (Fig. 4A). The results of GO enrichment showed that response to xenobiotic stimulus, response to hypoxia, response to decreased oxygen levels, response to lipopolysaccharide, and response to oxygen levels were the most essential biological progresses in the treatment of osteoporosis by FZD. The results of GO showed that membrane raft, membrane microdomain, organelle outer membrane, outer membrane and mitochondrial outer membrane were the most significant cellular components. And heme binding, tetrapyrrole binding, nuclear receptor activity, ligand–activated transcription factor activity, and oxidoreductase activity were the most important molecular functions in the treatment of osteoporosis by FZD. It was reported that oxidative stress was closely associated with oxidoreductase activity and the metabolism of oxygen [23, 24]. Therefore, the results of GO analysis indicated that oxidative stress could be a potential target in the treatment of osteoporosis by FZD. In addition, the KEGG analysis was used to clarify the pathways associated with the targets. The results revealed that 99 pathways were associated with the common targets (Additional file 5). The top 30 pathways were presented in the diagram of KEGG enrichment analysis (Fig. 4B). The results of KEGG analysis showed that the NF-κB signaling pathway and the reactive oxygen species (ROS) were closely associated with the role of FZD in the treatment of osteoporosis. And details of both the NF-kappa B signaling pathway (hsa04064) and the reactive oxygen species (hsa05208) were presented in the Additional file 6.

### Molecular docking

**Fig. 5:**
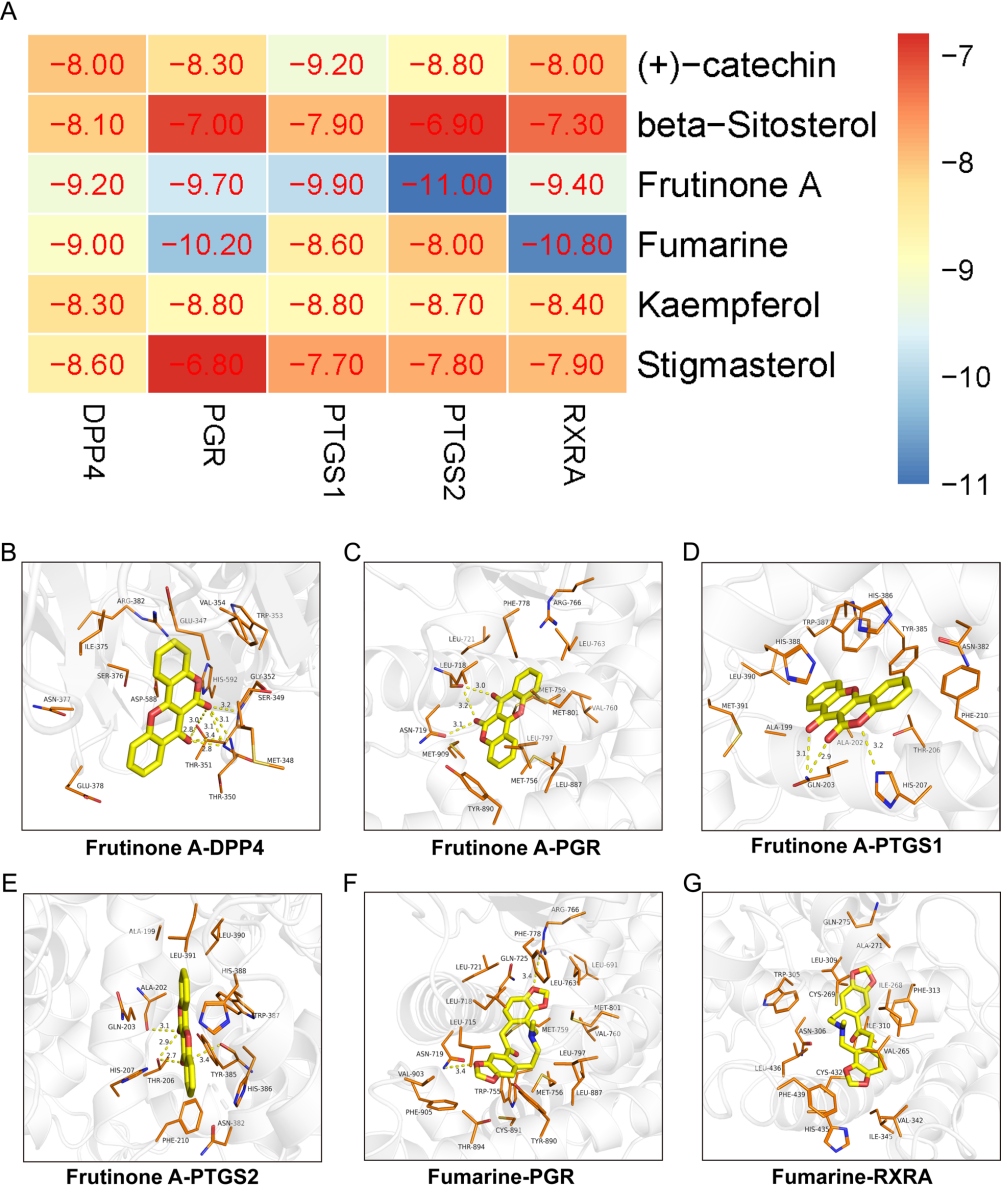
Molecular docking results. **A** Heat map of the binding energies. **B**–**G** The visualized molecular docking results

To further elucidate the potential mechanism through which FZD treats osteoporosis, molecular docking among ingredients and targets was applied. The six core bioactive ingredients (Frutinone A, Kaempferol, Stigmasterol, Fumarine, beta-sitosterol, and (+)-catechin) and the five core targets (PTGS1, PTGS2, PGR, RXRA, and DPP4) were obtained through the degree and the MCC methods. The binding energy was used to evaluate binding affinity among the components and the proteins. The results of molecular docking reveal that the binding energies among these core ingredients and core targets were all less than − 7 kcal mol^−1^, indicating that the compounds bind tightly with the protein targets (Fig. 5A). According to the binding energies, Frutinone A had the most rigid binding with DDP4, PTGS1, PTGS2, and PGR, and Fumarine showed the tightest binding with PGR and RXRA. And the molecular docking results of Frutinone A versus DDP4 (− 9.20 kcal mol^−1^), Frutinone A versus PGR (− 9.70 kcal mol^−1^), Frutinone A versus PTGS1 (-9.90 kcal mol^−1^), Frutinone A versus PTGS2 (− 11.00 kcal mol^−1^), Fumarine versus PGR (− 10.20 kcal mol^−1^), Fumarine versus RXRA (− 10.80 kcal mol^−1^), were visualized (Fig. 5B–G).

### Validation of the biological effect of FZD on the OVX-induced osteoporosis in rats

**Fig. 6:**
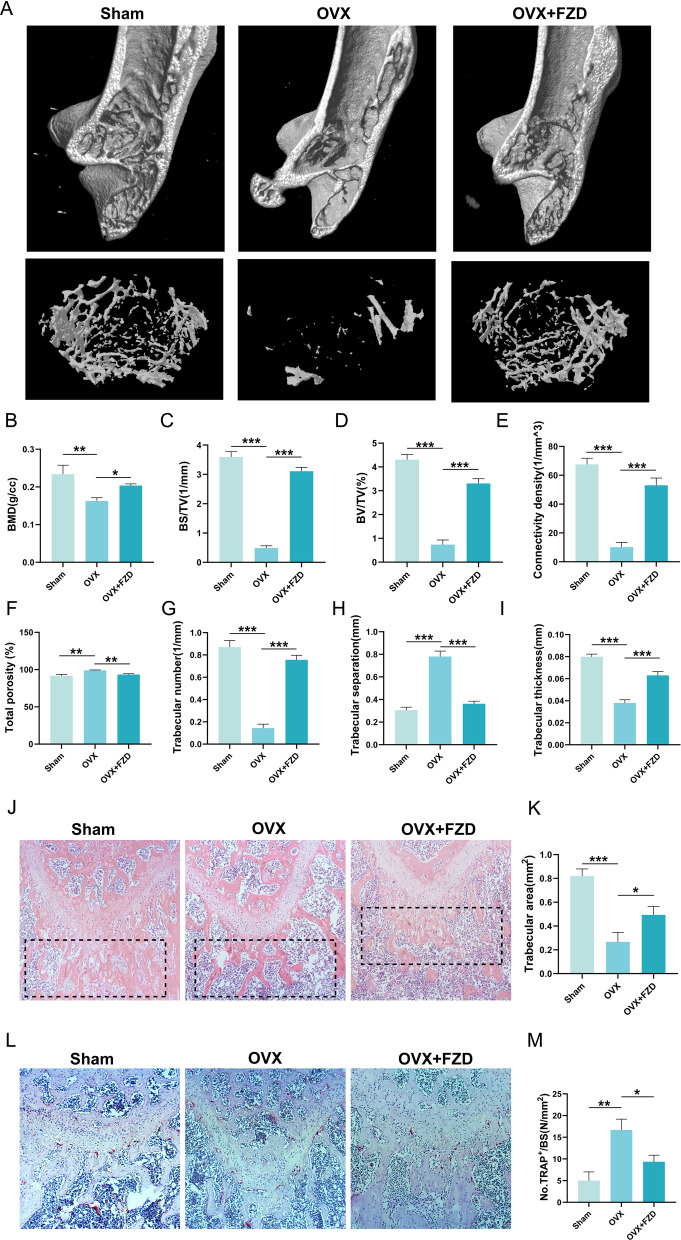
The biological effect of FZD on the OVX-induced osteoporosis in rats. **A** The images of micro-CT. **B**–**I** The quantitative analysis of Micro-CT results. **J**, **K** The H&E staining of the femurs and the quantitative analysis results. **L**, **M** The results of TRAP staining and the quantitative analysis results. NS indicates no significance. Values are the mean ± SD. *p < 0.05, **p < 0.01, ***p < 0.001

To verify whether FZD could exert protective effects on osteoporosis, the OVX-induced osteoporosis model in rats was applied. The microstructure of femur trabecular bone was analyzed by micro-CT. The images of micro-CT showed that the trabecular bone was sparse and thin in the OVX group when compared with that in the sham group, indicating that the OVX model in rats was reliable (Fig. 6A). The parameters of the trabecular bone were analyzed. The BMD in the OVX group was dramatically lowered, which was significantly increased in the OVX rats treated by FZD (Fig. 6B). The decreased BS/TV and BV/TV induced by the OVX were remarkably raised in the OVX + FZD group, indicating that the bone surface density and the relative bone volume were restored by FZD treatment (Fig. 6C, D). In addition, the decrease in the connectivity density, trabecular number, and trabecular thickness was ameliorated (Fig. 6E, G, I). And the total porosity and trabecular separation were increased after the OVX procedure, which were alleviated by FZD (Fig. 6F, H). H&E staining of the femurs demonstrated that the trabecular area in OVX rats was dramatically lowered, whereas the area of trabecular bone in OVX rats treated with FZD was significantly restored (Fig. 6J, K). What’s more, the TRAP staining showed that the TRAP^+^ OCs were red and distributed along the trabecular bone (Fig. 6L). The number of TRAP + OCs was lifted in the OVX rats, which was significantly decreased after the treatment of FZD (Fig. 6L, M). The results above demonstrated that the OVX-induced bone loss could be ameliorated by the treatment of FZD.

### The CCK-8 result and the anti-apoptosis effect of FCS on BMMs

**Fig. 7:**
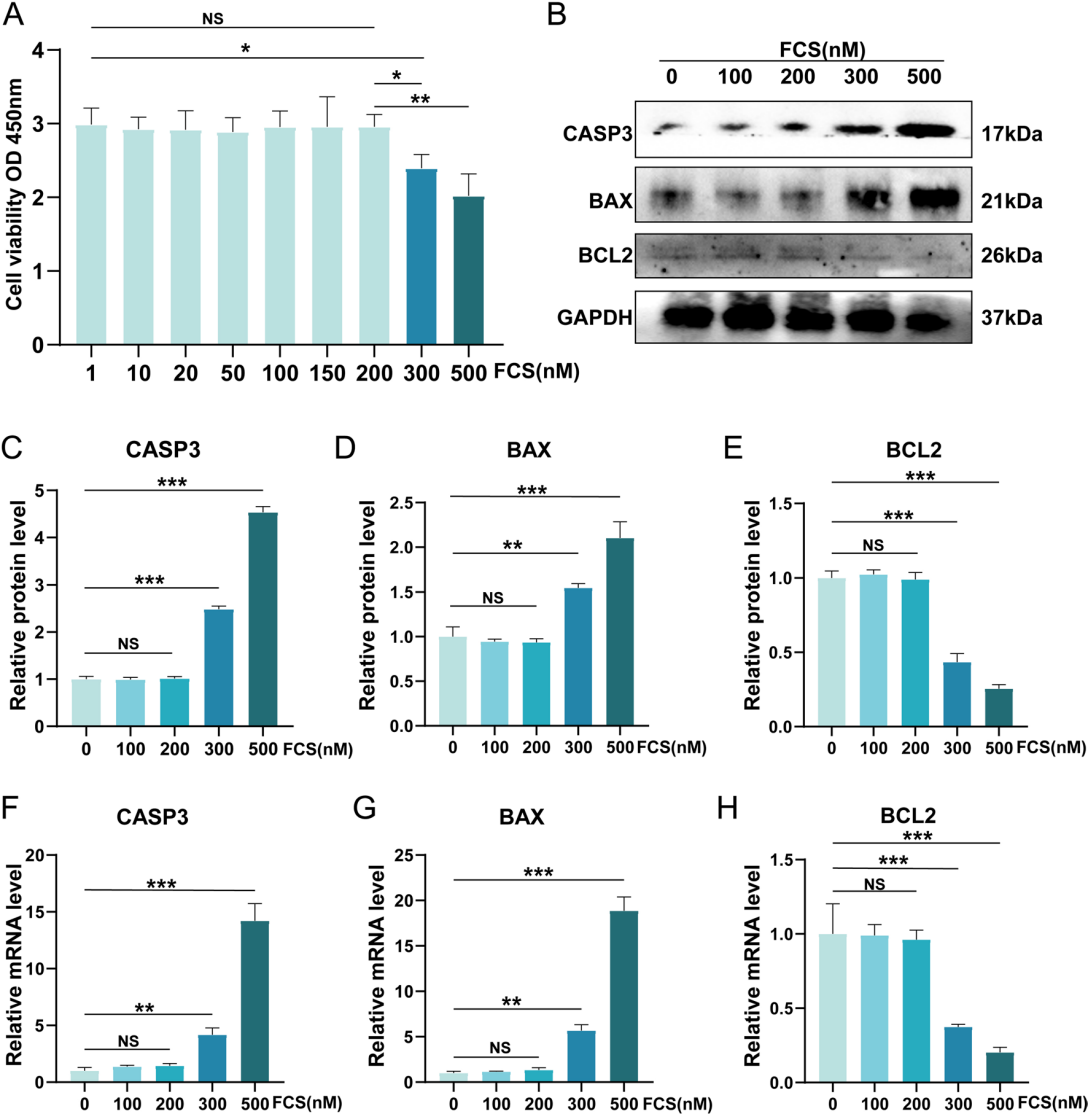
The CCK-8 result and the anti-apoptosis effect of FCS on BMMs. **A** FCS not more than 200 nM could not negatively affect the cell viability of BMMs. **B** The western blot results of the apoptosis-related indicators including CASP3, BAX, and BCL2. **C**–**E** Semi-quantitative analysis of western blot results on the expression of CASP3, BAX, and BCL2. **F**–**H** Semi-quantitative analysis of the mRNA levels of CASP3, BAX, and BCL2. NS indicates no significance. Values are the mean ± SD. *p < 0.05, **p < 0.01, ***p < 0.001

The cell viability of BMMs was detected using the CCK-8 method, indicating that FCS not more than 200 nM showed no cytotoxicity to BMMs (Fig. 7A). In addition, the levels of apoptosis-associated markers including CASP3, BAX, and BCL2 were detected. Similarly, the protein and the mRNA levels of the pro-apoptotic factors including BAX and CASP3 were not significantly increased when BMMs were treated with FCS not more than 200 nM (Fig. 7B, C, D, F, G), and the protein and mRNA levels of the anti-apoptotic BCL2 were not reduced when FCS was not more than 200 nM (Fig. 7B, E, H). The above results demonstrated that the FCS not more than 200 nM showed no cytotoxicity to BMMs.

### FCS inhibited RANKL-induced osteoclastogenesis and F-actin ring formation in a concentration-dependent method

**Fig. 8:**
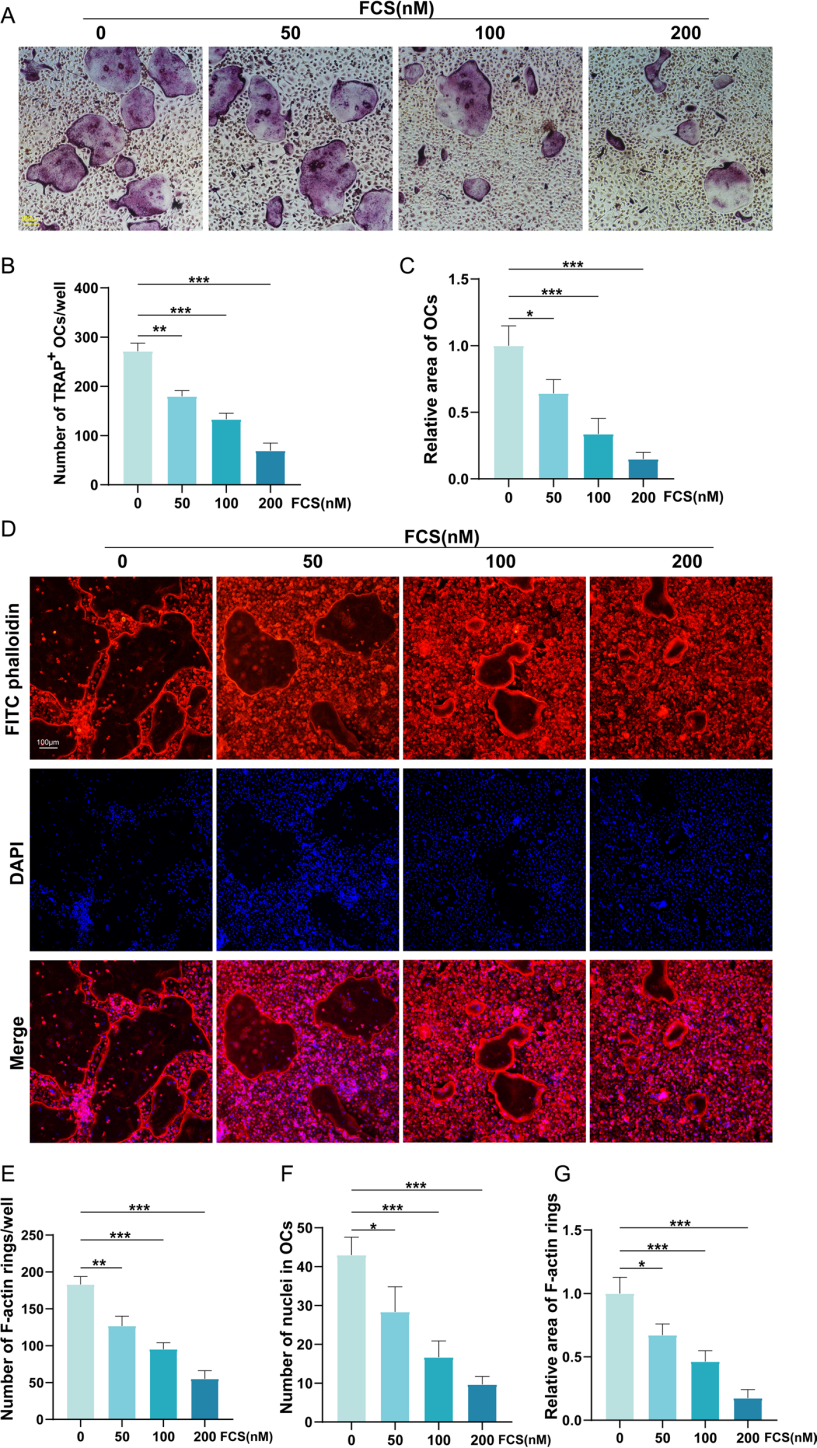
The results of RANKL-induced osteoclastogenesis and the formation of F-actin ring. **A** The images of TRAP staining. **B** The quantitative analysis results of the number of TRAP^+^ OCs in per well. **C** The semi-quantitative analysis results of the area of OCs. **D** The images of the F-actin ring of the RANKL-induced OCs. **E** The quantitative analysis results of the number of F-actin rings in per well. **F** The quantitative analysis results of the number of nuclei in OCs. **G** The semi-quantitative analysis results of the area of F-actin rings. Values are the mean ± SD. *p < 0.05, **p < 0.01, ***p < 0.001

Abnormal osteoclastogenesis tends to result in pathologic bone loss. To confirm the role of FZD in treating osteoporosis, TRAP staining was carried out. The TRAP staining of RANKL-induced OCs showed that the number of TRAP^+^ OCs in per well was dramatically decreased by the treatment of FCS in a concentration-dependent method (Fig. 8A, B). And the relative area of OCs was also lowered by the treatment of FCS (Fig. 8A, C). F-actin ring is a characteristic of mature osteoclasts, and it is considered as a critical indicator of bone-resorbing activity of OCs [25]. To further validate that the FCS suppressed the osteoclastogenesis, the F-actin ring in the RANKL-induced OCs was analyzed using phalloidin staining. The results of phalloidin staining demonstrated that BMMs treated with various concentrations of FCS formed significantly fewer number of F-actin rings than BMMs that were not treated with FCS (Fig. 8D, E). Consistently, the number of nuclei in OCs and the relative area of F-actin rings were also lowered by FCS in a concentration-dependent manner (Fig. 8D, F, G).

### FCS suppressed the RANKL-mediated expression of osteoclastogenesis-associated indicators

**Fig. 9:**
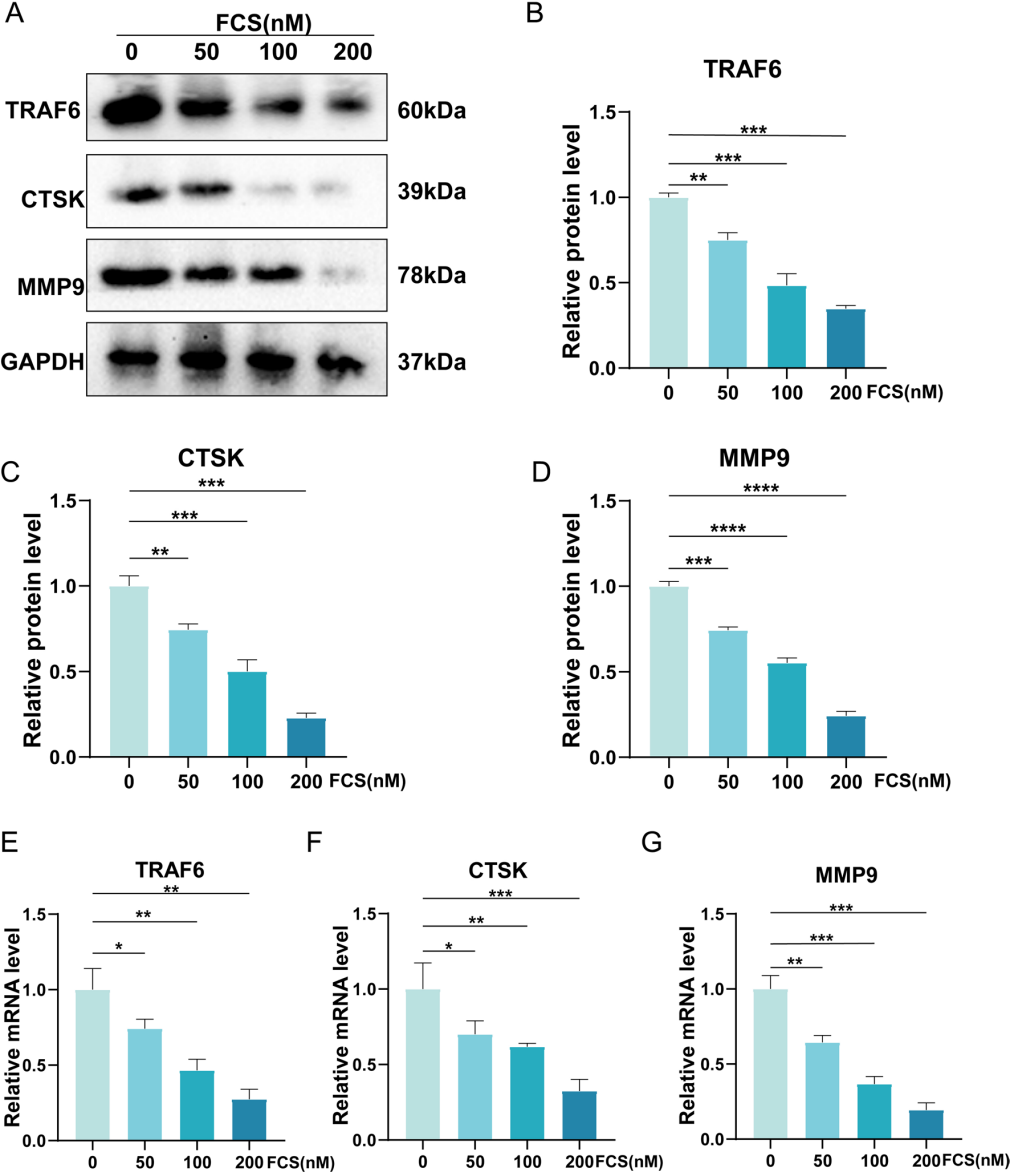
The effect of FCS on the expression levels of osteoclastogenesis-associated indicators. **A** The western blot results of TARF6, CTSK and MMP9. **B**–**D** The semi-quantitative analysis results of the protein levels of TARF6, CTSK and MMP9. **E**–**G** The semi-quantitative analysis results of the mRNA levels of TARF6, CTSK and MMP9. Values are the mean ± SD. *p < 0.05, **p < 0.01, ***p < 0.001

Tumor necrosis factor receptor-associated factor-6 (TRAF6) has been proved to be crucial for the activation of receptor activator of nuclear factor-κB (RANK) in OCs [26], which could help promote the differentiation of OCs. Cathepsin K (CTSK) and matrix metalloproteinase-9 (MMP9) have been reported to be secreted by osteoclasts to promote the degradation of the matrix during bone resorption [17, 27]. The above markers were used as indicators of osteoclastogenesis [17, 26]. To further explore the potential molecular mechanism underlying the inhibitory effect of FCS on OCs, the expression levels of TRAF6, CTSK and MMP9 were detected. The protein levels of TARF6, CTSK and MMP9 were all dramatically reduced in a concentration-dependent manner after BMMs were treated by FCS (Fig. 9A–D). Consistently, the mRNA levels of TARF6, CTSK and MMP9 were also decreased after the FCS treatment (Fig. 9E–G).

### FCS ameliorated the RANKL-induced activation of NF-κB pathway and the downstream factors essential for the formation of OCs

**Fig. 10:**
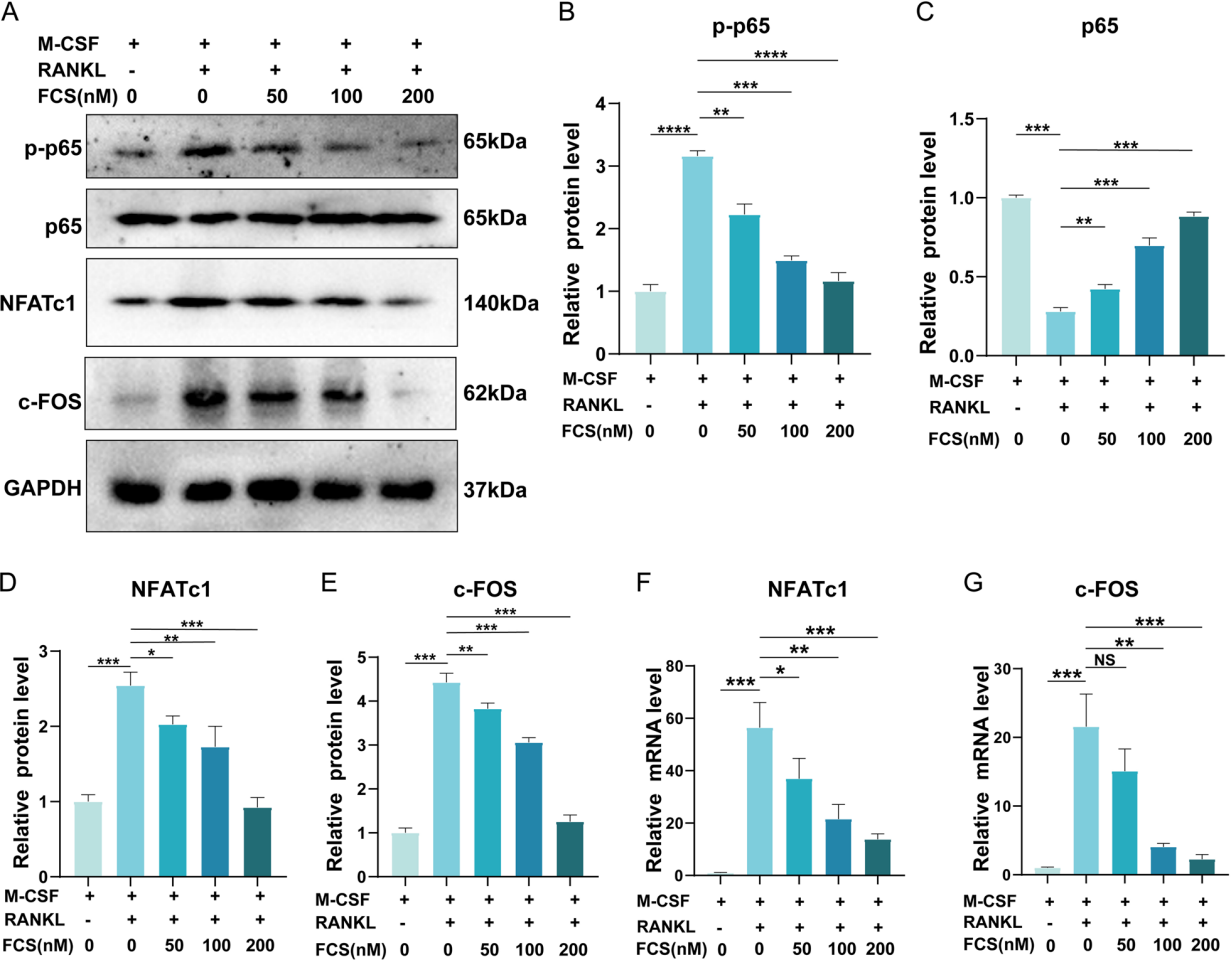
The RANKL-induced activation of NF-κB pathway and the downstream factors were suppressed by FZD. **A** The western blot results of p-p65, p65, NFATc1 and c-FOS. **B**–**E** The semi-quantitative analysis results of the protein levels of p-p65, p65, NFATc1 and c-FOS. **F**–**G** The semi-quantitative analysis results of the mRNA levels of NFATc1 and c-FOS. Values are the mean ± SD. *p < 0.05, **p < 0.01, ***p < 0.001

According to the results of KEGG enrichment results, NF-κB pathway was involved in the role of FZD against osteoporosis. And it was reported that the formation of OCs was inhibited by aspirin through regulating the NF-κB and Nuclear factor of activated T-cells cytoplasmic 1 (NFATc1) pathway [28]. What’s more, bone loss was ameliorated through suppressing the activation of NF-κB pathway and the expression of c-FOS and NFATc1 [29]. Therefore, the expression of NF-κB pathway-associated marker and the downstream factors c-FOS and NFATc1 were detected. The protein level of p-p65 in RANKL and M-CSF-treated BMMs, which has been reported to be one of the most important indicators of NF-κB pathway activation, was significantly increased, when compared with that in BMMs only treated with M-CSF (Fig. 10A, B). The protein level of p65 was decreased by the RANKL treatment (Fig. 10A, C). The results above demonstrated that the NF-κB signaling pathway was activated by RANKL and that the NF-κB pathway was involved in the OCs formation. What’s more, the increased protein level of p-p65 caused by RANKL treatment was significantly lowered by the treatment of FCS in a concentration-dependent manner (Fig. 10A, B). And the decreased protein level of p65 was markedly raised by FCS (Fig. 10A, C). The results indicated that FCS treatment could suppress the activation of the NF-κB pathway during the formation of OCs.

Then the expression levels of the downstream factors including c-FOS and NFATc1 were evaluated. The western blot results showed that the expression of both c-FOS and NFATc1 were raised after the induction of OCs through the treatment of RANKL (Fig. 10A, D, E), indicating that c-FOS and NFATc1 were indeed involved in the formation of OCs. The western blot results showed that the levels of NFATc1 and c-FOS were dramatically raised by RANKL, which were significantly decreased by the treatment of FCS in a concentration-dependent manner (Fig. 10A, D, E). In parallel with the western blot results, the increased mRNA levels of both NFATc1 and c-FOS induced by RANKL were significantly lowered by FCS (Fig. 10F, G). The above results demonstrated that the expression of OCs formation-related indicators, NFATc1 and c-FOS, could be suppressed by FCS.

## Discussion

Osteoporosis, a common condition in the elderly, severely comprises the quality of life and has exerted tremendous burden on the society and families. Investigating the underlying mechanisms of osteoporosis will be greatly meaningful. Bone homeostasis is dependent on the balance between osteoblasts and osteoclasts. The disequilibrium between the activities of osteoblasts and osteoclasts tends to be closely associated with bone loss, which can be caused by the overactivation of osteoclasts. Therefore, suppressing the excessive differentiation of OCs is an important treatment strategy. At present, an increasing number of medicines aiming at inhibiting the excessive formation of OCs have been used to treat osteoporosis, such as cathepsin K inhibitors and alendronate [30, 31]. However, a series of side effects resulted from these strategies have been reported, including neurobehavioral disorders and gastrointestinal side effect [32, 33]. FZD, one kind of traditional Chinese compound medicines, is characterized by good therapeutic effects and fewer toxic side effects and has been reported to be effective in treating osteoarthritis through ameliorating the degradation of extracellular matrix in cartilage [16]. And it was reported that the development of osteoporosis was also associated with the degradation of extracellular matrix caused by the MMP9 that could be secreted from OCs [34]. However, until now, no study has assessed the effects of FZD on osteoporosis. This present study was the first one to explore whether FZD could exert protective effects on osteoporosis through suppressing RANKL-induced OCs differentiation.

Based on network pharmacology, core targets (PTGS1 and PTGS2) and key ingredients (kaempferol, beta-sitosterol, stigmasterol, fumarine and frutinone A) that might play pivotal roles in treating osteoporosis were screened out. Previously reported study demonstrated that the decreased osteoclastogenesis might be associated with the expression of PTGS2 (also known as COX2) [35]. And several studies reported that the inhibition of osteoclastogenesis and resorption activity of mature OCs could be achieved through inhibiting the production of COX2 and prostaglandin E_2_ [36, 37]. In addition, it was revealed that the expression level of PTGS1 (also known as COX1) was upregulated in valve interstitial cells isolated from calcified tissues, and that the PTGS1 level in normal valve interstitial cells was raised by stimulation with osteoblast differentiation medium [38]. Therefore, PTGS1 and PTGS2 might be involved in the development and treatment of osteoporosis, and our results of network pharmacology were in line with previous studies. In our study, the direct effects of FZD on PTGS1 or PTGS2 were not explored, and this topic deserves to be explored in future studies.

As for the key ingredients of FZD in treating osteoporosis, a series of studies have been conducted to explore the roles and potential mechanisms involved in the treatment of diseases via these ingredients. Kaempferol that presents in commonly used traditional Chinese medicines such as Ginkgo biloba, Moringa oleifera and propolis, is the first leading component of the key ingredients of FZD in this present study. Previous studies revealed that kaempferol could exert potent osteoprotective effects through complex and multifaceted mechanisms. For example, Trivedi et al. found that kaempferol prevented the femur and the vertebra from OVX-induced trabecular bone loss. And the BMMs isolated from the kaempferol-treated OVX animals had more mineralized nodules when compared with the BMMs from the control group [39]. Prouillet et al. [40] revealed that the alkaline phosphatase activity in MG-63 osteoblasts was increased by kaempferol, which could also be suppressed by extracellular-regulated kinase (ERK) pathway inhibitor and estrogen receptor antagonist. Their findings demonstrated that the increased alkaline phosphatase activity was induced by kaempferol via activating the ERK signaling pathway that has been proved to be the downstream target of estrogen receptor activation [40]. In addition, Chang-Ju and colleagues revealed that kaempferol inhibited the osteoclastogenesis through regulating the autophagy process in RAW 264.7 cells [41]. Therefore, previous studies demonstrated that kaempferol could help ameliorate osteoporosis through complex mechanisms. In our present study, the results of network pharmacology demonstrated that kaempferol was closely related to PTGS1 and PTGS2, indicating that kaempferol might exert inhibitory effect on the osteoclastogenesis through regulating the expression of PTGS1 and PTGS2. Besides kaempferol, other key ingredients in FZD such as beta-sitosterol and stigmasterol have also been reported to have osteoprotective effects [42, 43]. These core components of FZD might be responsible for the therapeutic effects of FZD on retarding the development of OVX-induced osteoporosis. This present study revealed the protective effects of the forum, but the role of each ingredient and the involved mechanisms were not explored. Based on this study, further researches remain to be conducted.

According to the experiments in this present study, FZD retarded the process of osteoporosis via suppressing the maturation of multinucleated OCs. The expression levels of the OCs-related indicators such as TRAF6, CTSK and MMP9 were lowered by FZD. TRAF6 has been proved to be essential for the activation of RANK that plays an important role in the differentiation and function of OCs [26]. And previous studies demonstrated that the LPS-induced osteoclastogenesis could be inhibited by artesunate through suppressing the TRAF6 expression [44]. Consistent with previously reported findings, data in our present study demonstrated that RANKL-induced TRAF6 was reduced by the treatment of FZD in a concentration-dependent manner, revealing that the differentiation of OCs could be inhibited by FZD through regulating the expression of TRAF6. To degrade the extracellular matrix, both CTSK and MMP9 were produced and secreted by OCs during bone resorption [17, 27]. In our study, these osteoclastogenesis-associated indicators were lowered by FZD, indicating that FZD could ameliorate the osteoporosis through regulating the expression of the above OCs-related markers. In addition, the KEGG enrichment revealed that the NF-κB signaling pathway was one of the top leading pathways involved in the FZD-induced treatment of osteoporosis. This was in line with previous studies. It was reported that the RANKL-induced OCs differentiation was ameliorated by aconine through inhibiting the activation of the NF-κB signaling pathway and the downstream NFATc1 [45], indicating that the NF-κB pathway played an indispensable role in the maturation of OCs and the development of osteoporosis. Consistently, the results in our present study revealed that the NF-κB pathway was activated by RANKL treatment, which demonstrated that the NF-κB pathway was indeed involved in the formation of OCs. In addition, the activated the NF-κB pathway was inhibited by FZD. These results indicated that FZD could alleviate the osteoporosis through modulating the NF-κB pathway.

The present study also has several limitations. Although the NF-κB signaling pathway was indeed regulated by FZD, rescue experiment should be carried out to further validate the role of the NF-κB pathway in retarding osteoporosis and this will be carried out in future studies. Many other predicted signaling pathways or targets were not identified and further researches will be conducted in the future.

## Conclusions

Collectively, the present study identified several core targets, ingredients and signaling pathways that might be closely associated with the therapeutic role of FZD in countering osteoporosis. Based on network pharmacology and experimental validation, this study provided insights into the potential mechanisms in the treatment of osteoporosis by FZD. The results revealed that FZD alleviated the OVX-induced bone loss. And FZD downregulated the expression of osteoclast function-associated genes including TRAF6, CTSK and MMP9. In addition, the RANKL treatment-induced activation of the NF-κB pathway and the downstream NFATc1 and c-FOS was inhibited by FZD, which demonstrated that FZD could exert inhibitory effects on the formation of OCs and bone resorption through regulating the NF-κB pathway. Taken together, the findings of this study suggested that FZD could be a strategy for the treatment of osteoporosis. This study laid a good theoretical foundation for further investigations.

## Supplementary Information

**Additional file 1.** The active components of FZD.

**Additional file 2.** The targets of the herbs.

**Additional file 3.** The gene targets of osteoporosis.

**Additional file 4.** The common targets between FZD and osteoporosis.

**Additional file 5.** The pathways associated with the common targets.

**Additional file 6.** The details of both the NF−kappa B signaling pathway and the reactive oxygen species.

## Data Availability

All data generated or analyzed during this study are included in this article.

## Abbreviations

OBs: Osteoblasts
OCs: Osteoclasts
TCM: Traditional Chinese medicine
FZD: Fuzi decoction
OVX: Ovariectomy
MMP: Matrix metalloproteinase
TCMSP: Traditional Chinese Medicine Systems Pharmacology Database and Analysis Platform
OB: Oral bioavailability
DL: Drug-likeness
OMIM: Online Mendelian Inheritance in Man
TTD: Therapeutic target database
GO: Gene ontology
KEGG: Kyoto encyclopedia of genes and genomes
BMMs: Bone marrow-derived macrophages
PBS: Phosphate buffer solution
α-MEM: Alpha-minimum essential medium
RANKL: Receptor activator of NF-κB ligand
RANK: Receptor activator of nuclear factor-κB
CCK-8: Cell counting kit-8
OD: Absorbance density
Micro-CT: Micro-computed tomography
BMD: Bone mineral density
BV: Bone volume
TV: Tissue volume
BS: Bone surface
TRAP: Tartrate-resistant acid phosphatase
PPI: Protein–protein interaction
BP: Biological progress
CC: Cellular components
MF: Molecular functions
TRAF6: Tumor necrosis factor receptor-associated factor-6
CTSK: Cathepsin K
MMP: Matrix metalloproteinase
MMP9: Matrix metalloproteinase-9
NFATc1: Nuclear factor of activated T-cells cytoplasmic 1
FCS: FZD-containing serum
