# Supplementary figures and images for: Exploring the role and mechanism of Fuzi decoction in the treatment of osteoporosis by integrating network pharmacology and experimental verification
Source: J Orthop Surg Res. 2023 Jul 18;18:508. doi: 10.1186/s13018-023-03842-1 (PMC10354906; doi:10.1186/s13018-023-03842-1)

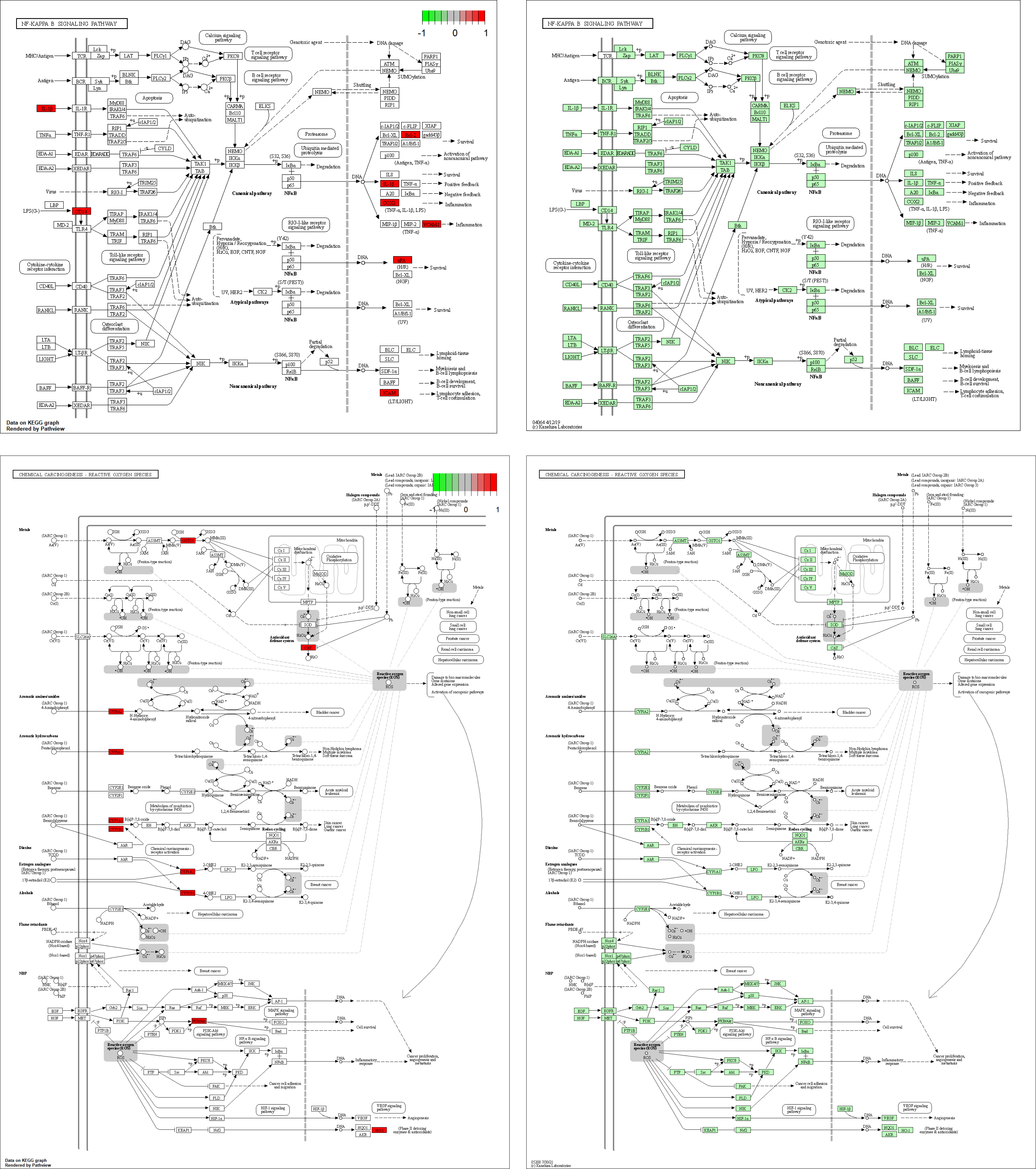

Supplement: Supplementary file 6 — Additional file 6. The details of both the NF−kappa B signaling pathway and the reactive oxygen species. [file 13018_2023_3842_MOESM6_ESM.jpg]
